# The Influence of Modern Physical Education of Females, in Producing and Confirming Diseases of the Spine

**Published:** 1830

**Authors:** 


*The influence of Modern Physical Education of Females in producing and confirming Deformity of the Spine.* By E. M Duffin, Surgeon. New-York, Chas. S. Francis, 1830. pp. 140.

We have selected this little work, on the present occasion, on account of the great importance which the subject of which it treats is deriving from the increased frequency with which these diseases recur, in consequence of the rapid advances which our society is making in refinement and luxury. No species of bodily deformity is more frequently met with, at least in the upper walks of life, than curvature of the spine. In addition to the tormenting reflections which are perpetually haunting the unhappy subject of every species of physical deformity, this particular form is accompanied with many unpleasant associations, which makes it peculiarly distressing to the female mind ; and what should give the subject additional interest, it is most likely to fall upon those whose quick perceptions, refined sentiments, and cultivated minds, render them peculiarly sensitive to the real or imaginary prejudice which results from it.

The subject is one with which every parent and every instructor of youth, should be familiarly acquainted, for the evil is so insidious in its growth, that it generally assumes a very considerable degree of importance, before the anxieties of the parents are sufficiently excited to call for the advice of the family physician. For these considerations we have selected this work with a view of directing the public attention to an evil which is daily acquiring additional importance, and which must necessarily be very imperfectly guarded against, if the prevention, as well as the remedy, be entirely entrusted to the medical profession.

In the 1st and 2d chapters, the author treats of those causes of deformity which grow out of sentiments and habits that are almost necessarily connected with a state of social refinement. He denies that permanent inclination of the spine necessarily depends upon original debility of constitution, or upon active and manifest disease, although certain constitutional and hereditary taints favor its formation very much. In the absence of such constitutional debility, it may be traced to sedentary and erroneous habits, "to an injudicious system of school discipline, to neglect or actual mismanagement of the physical education of young females, to the vitiated state of public taste, with respect to the perfection of female attire, and to many absurd customs and useless restraints of society, derived from the vain and ludicrous efforts of modern fashion to attain the very highest point of refinement."

Females are peculiarly liable to diseases of the spine, in consequence of the delicacy of their structure and constitution. This tendency is still further increased by the sedentary habits to which the sex are necessarily confined. The early period of life at which females are generally married, requires that their education shouldbe completed within a period of time very short, in comparison with what is devoted to that of young men.

The increased diffusion of knowledge which distinguishes the present age, increases the quantity of information to be expected from a young lady of finished education, while the tendency of fashion and refinement tends inevitably, to abridge very materially, the period for its acquisition. The late hours of rising so common in fashionable life, the mistaken tenderness of mothers, which prevents them from adopting or enforcing a systematic system of instruction, the minute attention to fashion which descends to the regulation of the most trifling regulations of dress and deportment, with an anxiety very frequently proportionate to their insignificance, are so many circumstances calculated to divert the attention of young females from more serious studies, and to require them to encroach upon those hours which ought to be devoted to exercise and recreation.

"Lastly, much time," says our author, "is lost in female seminaries in unskilful attempts to prevent, or unavailing efforts to correct deformities of the person ;" a labour that at least may be said to be useless, since with a judicious system of physical education, few cases would occur, requiring any remedy but the use of natural means.

In addition to these general causes connected with the present state of society, there are others which tend more particularly to influence the modes of education, and thus to favor the formation of diseases of the spine. Of these, one of the most considerable is the great competition which is to be met with on the subject of teaching. Very few of the immense number of competitors for public favor in this department, are sufficiently acquainted with the principles of education, to superintend the education of youth, and of those who do, a still greater proportion are ignorant of the principles of physical discipline, or at least consider it as altogether a subject of minor consequence.

The estimate formed by the public of the qualifications of a teacher, is generally superficial, and is founded rather upon the amount of facts, or of words committed to memory, than upon the store of useful ideas, or the formation of those habits of thinking which are the true object of education. With the full knowledge of this erroneous impression on the part of the public, the first object of the great proportion of teachers is, to meet the expectations of parents and friends; and to effect this, children are subjected to a confinement perfectly incompatible, either with bodily health, or with a vigorous exercise of the faculties of the mind; while to preserve the attention under a course so inconsistent with the dictates of nature, the feelings and passions are enlisted to a degree that is scarcely less injurious. Between the want of exercise, and the excessive mental excitement, the general health suffers, and a foundation is laid for a great variety of local diseases.

The following is the author's description of the most common deformity of the spine:

"The right shoulder projects more than is natural, and is, in common parlance, said to be 'growing out.' The course of the central groove of the back, deviates from a straight line; a greater distance is observed between a given point of the original perpendicular *spinal* line and the top of the elevated shoulder-bone, than between the same point and the corresponding top of that of the left side. These appearances, together with a remarkable prominence of the lower-third of the shoulder-blade of the distorted side, alarm the parents, now surprised at the extent to which the deformity has proceeded without having attracted much notice.

The gait of the young person appears awkward and shuffling ; her clothes cannot be made to sit well upon her—they appear to be drawn to the right side. The sash encircling her waist, is observed to dip towards the same side, while the right breast presents a remarkable fullness, and the corresponding collar bone displays a proportionate elevation.

In short, she is crooked; her back-bone is distorted. In a multitude of instances, even in this early and remediable stage, absolute and permanent deformity can be prevented only by care and attention of no ordinary kind, directed upon principles derived from a thorough knowledge of the nature of the parts affected. In proportion as the inclination takes place in the upper part of the back, between the shoulders, nature, in order to counterbalance the evil, and preserve the equilibrium of the body, calls into action the muscles of the lower part of the spine; these operate with proportionate power on the opposite side, so that, in confirmed cases, there is in fact a double curvature produced.

As the distortion advances, a similar counterbalancing power is exerted by the muscles attached to the spine in the neck, and a third, or *upper* curve, is then formed, so that the spine presents, in fact, a serpentine appearance, inclining to each side alternately. The ribs, in consequence of the alteration in the course of the spine, deviating from their true direction, partake of the change instituted. Finally, the basis, or *pelvis*, on which the spine rests, becoming involved, produces an inequality in the size of the hips, the contrary of that which presents itself in the shoulders, and causes the whole body, when viewed from behind, to appear as if twisted on itself."

When these symptoms are first observed, the child should be immediately removed from school, and a different system at once adopted. But unfortunately the first recourse is to the different modes of pressure by means of stays; &c., &c, which, so far from remedying the evil in question, superadd some other deformity, while the injudicious system of education is kept up until the evil becomes irreparable.

Chapter IV. gives a general idea of the mechanism of the spine, and of the influence of artificial modes of support in producing deformity.

The spinal column consists of 24 small bones, denominated vertebræ, containing a canal for the transmission of the spinal marrow. The bodies of these bones are united by a compressible, but very elastic substance, of a cartilaginous nature, and they are articulated by means of processes so constructed, as to admit a very considerable degree of motion in almost every direction. This column is maintained in the perpendicular direction, by the elasticity of the intervening cartilage, and by innumerable muscular fibres, arising from the bodies and processes of the vertebræ, and inserted into those above. So admirable is the adjustment of these muscles, and of this cartilaginous bond of union, that by the instinctive action of the one, and the mechanical operation of the other, the whole pile is kept perfectly upright, in regard to its lateral aspect.

After this general view, the author lays down two principles, from the consideration of which, we may understand how the circumstances already enumerated, may operate in producing deformity.

"Now it is a law of the animal economy, that whenever the natural and healthy operations of any organ, or set of organs, are either not regularly, or not sufficiently exercised, the organs whose operations are so disturbed or omitted, suffer material injury in a proportionate loss of their capabilities of action. In some instances, indeed, the derangement so produced gives rise to active disease.

Again, organs, when they are too much exerted, or when their natural operations are kept up beyond certain limits, become fatigued and incapacitated for the performance of their wonted office, until by repose they are enabled to obtain a renewal of their exhausted nervous or vital energy." p. 41.

Now the effect of the modern restraints of female attire is, to prevent the free action of the muscles of the spine, and by the artificial support thus given, to restrain, in a very considerable degree, the exercise of that instinctive power by which the balance of the body is preserved. The muscles thus not only lose their power of performing their natural actions, but frequently become wasted in their substance. Consequently, when the artificial support is removed, the individual complains of weakness, and of inability to support herself erect.—Under these circumstances, the sudden discontinuing the use of the stays, may tend to increase, rather than prevent the deformity produced by their use.

It is needless to refer to the injurious effect produced by tight stays upon the viscera. The consequences of their pressure upon the lungs, stomach, and other viscera, and the interruption of their functions which must ensue, are too obvious to require comment. We are not disposed to recommend their discontinuance. Fashion has rendered them necessary; but when they are used to improve the form, or indeed, with any other object than the simple adjustment of the dress, not only are they injurious, but they defeat the very object which leads to their employment.

In the 5th chapter the author inquires if unequal muscular action is ever sufficient of itself to produce deformity.

* * * "I have a case of lateral curvature before me, where the girl wore no corsets or other restraints of dress before she was fourteen years of age, lived till then in the country, and had full permission to roam about, almost at pleasure. This young person has a slight congenital, perhaps hereditary, deformity in the feet, to which circumstance I would entirely attribute the lateral curvature: for just as lateral curvature may produce a shuffling manner of walking, so a shuffling manner of walking may in time produce a lateral curvature. In like manner the advanced shoulder, and halting step of the ploughman, are to be attributed to one foot being always in the furrow, and to the inequality of the soil on which he is accustomed to walk. Sailors have generally the spine bent *forwards*, a characteristic which Smollett has not neglected to bestow upon that *beau ideal* of a British seaman, Commodore Trunnion. This stoop, or bend in the back, is most remarkable in men who serve on board the larger vessels of the line, owing to these vessels consisting of several decks, the height between which is not sufficient to permit a man of the ordinary stature to stand upright. It is notorious that artisans generally contract some bend or twist in their backbone or limbs, so characteristic as to enable a practised eye easily to judge of their respective pursuits without any other information than what is derived from their appearance. Clerks, and other sedentary persons, frequently contract the lateral, or twisted curvature. 'I have seen,' says Mr. Bamfield, 'instances of *lateral curvature* produced by a habit of long continued inclination of the body to one side, after the adult age, in insane persons; in the young and growing this is a more common event.—Young artists of both sexes are liable to lateral curvature from this cause, from adopting a habit of sitting before their paintings and drawings, with an inclination of the body to the left side.' * * * Dr Harrison informs us, that among the colliers of a particular mine in Lancashire, who are obliged, from the thinness of the stratum, to sit in a bent posture, and to force their right side into the vein, while digging out the coal, the spines of all of them become, in process of time, curved towards the right side." p. 59—61.

The most curious instance of this kind is found in the fact, that among the male inhabitants of London, the habit of giving the wall by the left, and taking it by the right shoulder, has given an obliquity to the trunk, by which they are easily distinguished from strangers.

In chapter VI. the author inquires into the changes which take place in the vertebræ themselves, favoring the production of deformity.

The spine, we before remarked, consists of 24 vertebræ, united by a compressible, elastic substance, admitting of a very considerable extent of motion. As we descend in the chain, and the weight to be supported increases, the vertebræ become larger, and the processes to which the muscles are attached longer, so as to enable them to act with more power.

In childhood, both the bones, and their intervening cartilages, are much softer than in after life; indeed, in childhood, the processes are attached to the bodies of the bones, by a cartilaginous bond of union, which gives way to a deposition of bone, as life advances, and a necessity for a firmer structure occurs.

This change of cartilage into bone is effected in the following manner. Small vessels conveying red blood are seen pervading the white cartilaginous substance which is absorbed in the vicinity of the artery, and its place supplied by a bony deposit from the arterial extremities. This supply of red blood, as it is uniformly present where the process of ossification is going on, would appear to be indispensable for that purpose. Now any one who has observed how defective is the formation of red blood, and how imperfect its distribution in the extreme vessels of those who devote themselves to hard study without sufficient exercise, will readily comprehend also how, under similar circumstances, the ossification of the spine will take place very imperfectly, and will at once discover how the restraint, excessive study, constrained positions, and the excitement of the passions in our female boarding schools, must favor the production of the disease in question. The following extract will show how this deformity usually takes place.

"The cartilages, ligaments, and muscles, being supplied with nourishment from the same source as the bones, suffer equally from the same privation. The muscles become not only more languid and feeble than they ought to be, but are sooner exhausted. The indications of lassitude, necessarily, from these causes, pervading the general carriage of the child, being attributed by the teacher to indolence, is attempted to be combated by a rigorous enjoinment of some particular, generally the erect posture. The suffering in consequence of this rigor soon becoming considerable, the child endeavours to render it tolerable by means of alternate efforts at balancing. A person seated upon a stool or chair, may throw the weight of the head, trunk, and upper extremities upon either of the hips, almost without any apparent deviation of the spine from the perpendicular. This is effected by drawing the spine to one side, and leaning the head and neck slightly to the other. I am persuaded that in this manner girls often rest themselves when writing, or when playing upon the piano and upon the harp, though they are thought to be sitting sufficiently upright. The right hand, being in all of these occupations that which requires most scope for motion, gives rise to the right shoulder being raised, and, in order to facilitate this, to the balance of the body being maintained on the left hip. The curvature that arises from these habits, is thus directed to the right side."

p. 76—77.

In chapter VII. the author offers some valuable reflections upon the subject of the education of children. Parents and teachers often seem to forget that "doubling the power does not double the effect;" that the mind like the body has its periods of futigue, and that if its exhausted powers are urged to still further exertion, so palpable a violation of the dictates of nature must be attended with the most injurious consequences.

The same mode of reasoning may apply to the mode of education. In childhood the judgment is weak, the curiosity ardent, and the memory st ong *when assisted by the imagination, or employed upon sensible objects.* These facts obviously point out the system of education proper for children of an early age, and would lead us to suspect that when their minds are occupied upon ideas with more complicated relations, the time is at least lost, if indeed the regular development of the mental faculties be not injured.

The author very properly cautions against the excessive cultivation of early talents. They are generally premature, and almost invariably, if fostered, lead to mental exertions that prevent the healthful development of the body, and frequently to deformity, despondency, or an early grave.

From the 9th chapter we select the following remarks on the subject of different modes of exercise :

" Dancing practised to excess, is very apt to injure the finer proportions of the limbs, by developing too fully certain of their parts more particularly called into action. Thus, in our best opera dancers, the ankle and calf of the leg are often clumsy and Herculean, compared with the slender, skinny arms of such persons. Professional dancers, moreover, are seldom remarkable for grace in any of the ordinary movements of life. In the performance of these they are generally constrained, formal, and automatic. The bad effects on the form of the foot, resulting from the force with which it is extended, gradually stretching its ligament beyond the point whence, even if possessed of more elasticity than they are, they can return, are well known. Very few opera dancers can boast of a good instep off the stage. When the foot is placed on the ground, the arch of the instep yields to the weight of the body, and allows the concave part of the sole to rest on the same plane with the toes. When, there fore, these persons walk, they never rise on the toe, nor bend the foot. From their habit of turning the toes very much outwards, they acquire a peculiar mode of walking, usually denominated 'a strut,' by some 'shailing,' a term more properly applied to walking sideways.

These observations apply to the bad tendency of dancing practised to excess, and are intended to point out how questionable is the propriety of devoting much time to this accomplishment, with a view to the attainment of more than graceful and natural habits of motion.

The young person wishing to use the dumb-bells with advantage, must stand perfectly erect, place the heels together, and point the toes slightly outwards. The bells being grasped one in each hand, must be raised simultaneously towards the front and centre of the chest, and approximated, so that the corresponding balls of each bell may touch each other respectively. The bells are now to be moved straight forwards, but not forcibly, to the full length of the arm, and, with the arms kept extended, allowed to drop with sufficient force to swing them gracefully round the body. The arms must be gently turned out in their course downwards, so as to make the balls on the outer or thumb side of each hand approximate or strike against each other behind the back, the elbow joint being kept as straight as possible. The bells are then to be again brought slowly round to the front and centre of the chest, and to be moved in the same manner for twenty, thirty, or any number of times that may be deemed necessary.

The usual fault in the mode of using the dumb-bells consists, either in not turning the arms outwards, as they are swung round the body, or in twisting them inwards. The fault in either case causes the child to elevate the shoulders in endeavoring to make the balls strike each other behind the back, and at the same time to thrust forwards the head and neck, and otherwise defeat the purpose for which the exercise is designed.

The weights may also be gradually raised at the full length of the arm from the sides, till such time as they are brought into contact above the head; then, the hands being turned outwards, allowed to fall slowly and steadily backwards, until the back of the hands meet, as in the former case.

Used in either of these manners, the dumb-bells are found a most efficient means of calling into salutary action all the muscles of the system. By their influence the chest is gradually and equally expanded, the shoulder bones are properly depressed and drawn towards the spine, and the symmetry of the back is otherwise improved in a remarkable manner.

Other exercises beneficial in their tendency, as walking on a rope not elevated more than fifteen or twenty inches above the ground, balancing such bodies of little weight, as a work-basket, pincushion, small bag of sand, and the like, upon the head, or a rod in each hand alternately, may be advantageously adopted as a part of school discipline for the prevention of this kind of deformity. These and similar amusements, being achievable only by an equal exertion of the muscles, at the same time that they tend to strengthen these organs, tend also to correct as well as prevent any deviation from the naturally erect form." p. 105—110

We shall close our review with an extract, pointing out the impropriety of attempting to correct deformity by the use of mechanical expedients which may have a tendency to produce irregular muscular contraction.

"If the shoulders be braced by means of straps to a plate of iron placed on the back, it is evident that the action of the muscles, with which nature has endowed the body for the express purpose of holding the shoulders in a graceful position, will be superceded, and, in accordance with the general law before enlarged upon, will, from want of due use, become proportionately incapable of performing their wonted office when the strap is removed. The muscles on the forepart of the chest, whose actions are destined as an equalizing and antagonist power, will, from being excited to resist the force exerted by the straps, become increased in strength. When the use of the straps is discontinued, the shoulders will not only be returned to the position which they held previously to the application of the plate, but be further drawn forwards by the power gained by the muscles on the forepart of the chest, while opposing the action of the straps. No constraining force, then, should be employed with a view to keep the shoulders back." p. 120—121.

We cannot close this review without recommending this little work to the attentive consideration of all entrusted with the education of youth. Its simplicity and perspicuity adapt it to every capacity. It should always be borne in mind, that the prevention of disease or deformity is far easier than their cure. An attention to the dictates of nature, which are so simple and perspicuous as never to be misunderstood, except by minds under the influence of prejudice or passion, is all that is requisite for the former. The latter is far more complicated, and requires an education proportionate to the progress of society in refinement and luxury. To this, it is the interest of the physician to confine himself; and to this it appears to be the settled determination of the public to confine him. For although the medical profession have at all times manifested a generous disposition to promote the interests of society, by pointing out the simple rules by which health is to be preserved, yet their instructions are always received with distrust, and if they venture to give them a particular application, an expression of suspicion very generally intimates that they are stepping beyond their province. Restricted, therefore, as we are in our attempts for promoting the interests of society, we are at least disposed to fulfil our duty by informing them, where the information may be acquired, which they are unwilling to learn from the physician; and where they may ascertain that disease and deformity, so far from being the work of nature, are acquired at an expense and labour, that is very rarely bestowed upon the acquisition of any other object, however valuable.

